# Delving into the innate and adaptive immunity of camelids: a paradigm of interspecies adaptation and evolutionary innovation

**DOI:** 10.3389/fimmu.2026.1833289

**Published:** 2026-06-24

**Authors:** Remya PG Ramesh, Avinash Premraj, Hadida Yasmin, Hind Alkaabi, Solaf Dodan, Maitha Almahri, Noura Alameri, Yaser Y. Alyaarbi, Khaja Mohteshamuddin, Ahmed Al Aiyan, Andrew J. T. George, Walter Conca, Uday Kishore

**Affiliations:** 1 Department of Veterinary Medicine, College of Agriculture and Veterinary Medicine (CAVM), United Arab Emirates University, Al Ain, United Arab Emirates; 2 College of Medicine and Health Sciences, United Arab Emirates University, Al Ain, United Arab Emirates; 3 Institute of Health Sciences, Presidency University, Kolkata, India; 4 Department of Surgery and Cancer, Imperial College, London, United Kingdom; 5 Department of Executive Health Medicine, King Faisal Specialist Hospital & Research Center, Riyadh, Saudi Arabia; 6 Department of Cell Biology, King Faisal Specialist Hospital & Research Center, Riyadh, Saudi Arabia; 7 Zayed Centre for Health Sciences, United Arab Emirates University, Al Ain, United Arab Emirates

**Keywords:** adaptive immunity, Arabian camel, camelids, *Camelus dromedarius*, dromedary camel, heavy chain-only antibodies, innate immunity, nanobodies

## Abstract

Camelids have evolved to survive some of the most challenging climatic conditions on our planet and are resilient to physiological stress such as temperature extremes, drought, storms, intense ultraviolet radiation, dehydration, and starvation. They exhibit remarkable resistance to several diseases that severely affect other livestock including tetanus, foot-and-mouth disease, and bovine spongiform encephalopathy. The robust nature of the camelid immune system, which maintains functionality under extreme conditions, is remarkable. The special features of the camelid immune system include differences (when compared to ruminants) in the distribution and anatomy of their lymphoid organs. The innate immune system merits further investigation, though potent antimicrobial peptides have been identified in camelid milk. It is in the adaptive immune response that the most distinctive aspects of the camelid immune system are seen, especially, the presence of heavy chain-only antibodies, somatic hypermutation of T cell receptors; in the dromedary, a significant number of γδ T cells. In this review, the interplay between innate and adaptive immunity in camelids is examined, highlighting their functions in systemic and mucosal immune defense. An understanding of these adaptations is important for the development of innovative biomedical applications, such as nanobody-based diagnostics and therapeutics. In addition, dromedary camels are the main likely animal reservoir for the Middle East Respiratory Syndrome Coronavirus (MERS-CoV), an emerging zoonotic pathogen with potential for large-scale human spillover. Finally, as climate change is likely to result in increased environmental stress worldwide, a comparison of how the immune system in different species has adapted to their environment will be important.

## Introduction

The extant members of the mammalian family Camelidae are represented by Old World (*Camelini*) and New World Camels (*Lamini*). The Old-World species include two-humped camels (*Camelus bactrianus* and *Camelus ferus*) and the dromedary or the Arabian camel (*Camelus dromedarius*) which is one-humped. New World camelids are represented by four species: the domestic llama (*Lama glama*), alpaca (*Vicugna pacos*), wild guanaco (*Lama guanicoe*) and vicuña (*Vicugna vicugna*). Old World camels have adapted to hot and arid desert environments, while New World camelids have evolved to thrive at high-altitude, and in the cold and arid Altiplano (the Andean Plateau). In addition to their elongated necks, high-quality wool, unique foot structure (extant tylopoda), humps (in camels), and three-chambered stomach camelids are particularly remarkable for their oval erythrocytes. Dromedary camels are known to host a wide range of pathogens, including viruses and bacteria that pose considerable risk to human health. In particular, dromedary camels are thought to be the primary reservoir for the zoonotic Middle East Respiratory Syndrome coronavirus (MERS-CoV), which causes MERS in humans with a high case fatality ratio (1). In contrast to the devastating progression of MERS-CoV infection in humans, dromedary camels manifest relatively mild and temporary respiratory symptoms, requiring little or no veterinary or clinical intervention. There are several potential factors underlying enhanced resistance of camels to MERS-CoV (2).

A robust immune system enables camels to resist several infectious diseases that severely affect other livestock such as tetanus, foot-and-mouth disease (FMD), and bovine spongiform encephalopathy (3). Dromedary camels have shown high resistance to the FMD Virus (FMDV) in experimental infections. Despite high-dose exposure to FMDV, dromedary camels did not develop any clinical signs of disease, viremia, or anti-FMDV antibodies (4). These attributes point to the adaptation of the dromedary camel immune system to such pathogens.

In addition to their adaptations to extreme environments, camelids have evolved atypical features in their adaptive immune systems, characterised by heavy chain-only antibodies and the use of somatic hypermutation in the T cell receptor (TCR) of their gamma delta (γδ) T cells (5, 6). The immune organs of dromedary camels are also morphologically and histologically different from ruminants, especially the thymus, tonsils, and Peyer’s patches (7). Despite the novel and distinctive features of their immune system, research on camel immunity has been limited. In this review, we aim to examine the current knowledge about the innate and adaptive immune systems of camelids, and reflect on their enormous potential as a unique model system for immunological studies.

## Peripheral blood cells

### Red blood cells (erythrocytes)

The particular oval shape of camelid red blood cells (RBCs) and their structural and physiological characteristics play a fundamental role in their remarkable resistance to osmotic lysis, reduced deformability, and enhanced affinity for oxygen. These characteristics are well-suited for existence in environments characterised by limited water resources in deserts or diminished oxygen levels at high altitudes. The erythroid membranes of both New World and Old World camelids contain higher levels of the integral membrane protein, band 3, in comparison to 16 other mammalian species (8). Furthermore, the enhanced cross-linking of band 3 with other membrane proteins in camelid RBCs probably contributes to their lower capacity to change shape and higher resistance to hypotonic lysis (9). While reduced RBC deformability may pose challenges for animals encountering diverse environmental pressures, this drawback is mitigated by the morphology and small size of camelid RBCs compared to humans, which facilitates their passage through capillaries without deformation. Even though the camelid RBCs are smaller in size, they can expand to double their volume, an advantage in harsh conditions (10). The dimensions and morphology of camelid RBCs enhance oxygen diffusion by offering an increased effective surface area for gas exchange. Another adaptation to life at high altitude and low oxygen tension is a leftward shift in the oxygen–hemoglobin dissociation curve, particularly at lower partial pressures, which enhances the uptake of oxygen by the lungs. Furthermore, the proportion of alkali-resistant (fetal) hemoglobin with enhanced oxygen affinity is greater in adult alpacas compared to other mammalian species (11). Electrophoretic analyses of hemoglobin have revealed that this pigment has two components, each showing minimal mobility. Lactate dehydrogenase is six times more abundant than in humans. The RBC glucose-6-phosphate dehydrogenase level is twice that of a person living at the same altitude (12).

**Figure 1:**
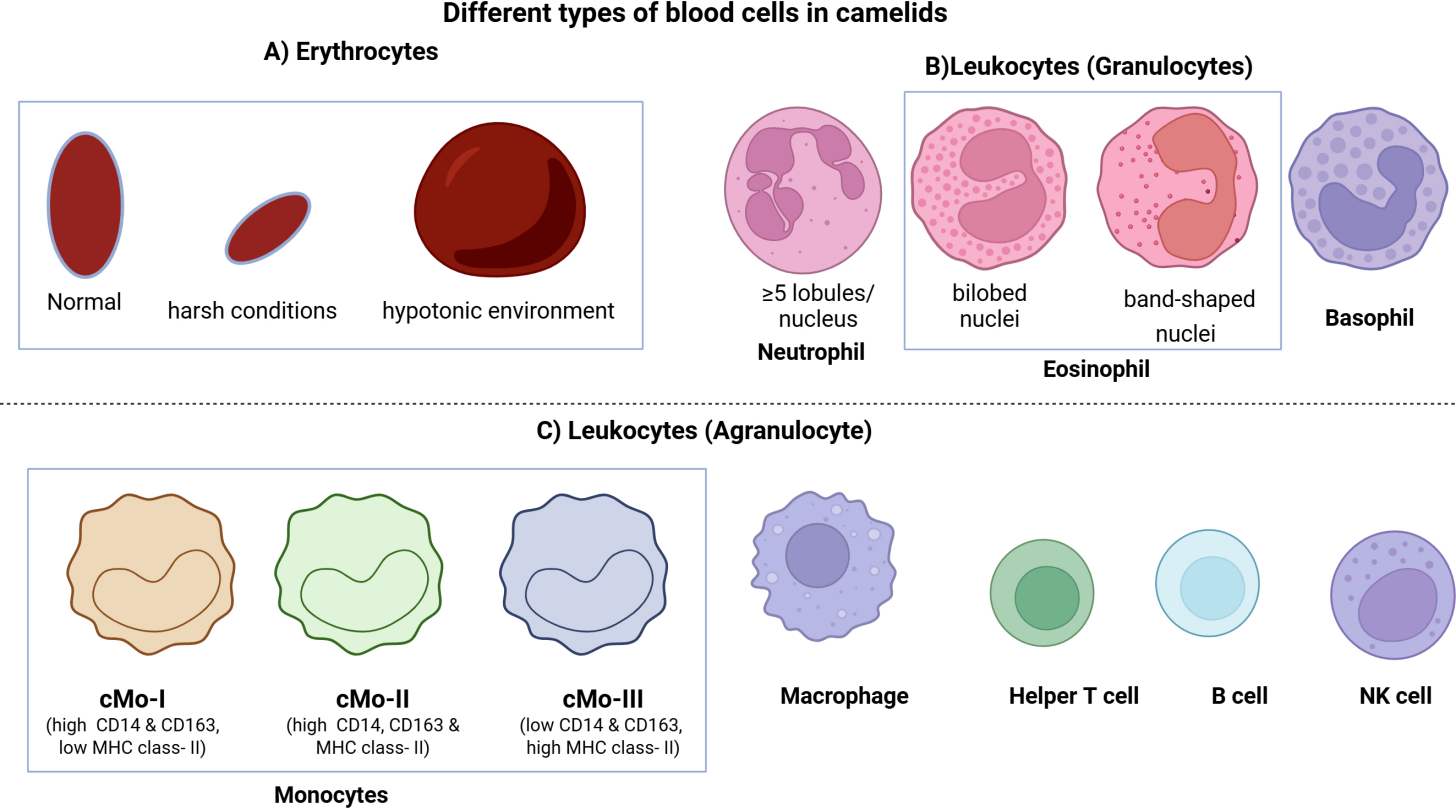
Illustration of blood cells in camelids. **(A)** Camelid erythrocytes are unique in structure and function. They exhibit exceptional resistance to osmotic pressure, reduced deformability, and enhanced affinity for oxygen. Camelid erythrocytes are oval and anucleate; their size can vary in harsh conditions. Even though they are small in normal conditions, they can expand to double their size. During harsh conditions, RBCs remain shrunken to resist dehydration. **(B)** Camelid leukocytes are like those of other mammals. Neutrophils are segmented. Eosinophils are characterised by band-shaped to bilobed nuclei. The cytoplasm contains numerous fine to small round eosinophilic granules within a cytoplasmic matrix. Basophils are recognised by their abundant granules, which may obscure the banded nucleus. **(C)** Monocytes share morphological characteristics with those of other species, including thick band nuclei and coarse chromatin. Camel monocyte cMo-I has high levels of CD14 and CD163 but low levels of MHC class II while cMo-II is characterised by high levels of CD14, CD163, and MHC class II. High levels of MHC class II but low levels of CD14 and CD163 are the peculiarities for the cMo-III. Macrophage cytoplasm contains abundant vacuoles and uneven cell edges.

The dromedary camel, living in arid deserts, shows tolerance to elevated temperature and possesses remarkable water conservation capabilities (13). The blood of camels is pivotal to this distinctive adaptation (14, 15) (**Figure 1A**). The blood cells of camels especially Old World camels, particularly RBCs, display notable changes in morphology, dimensions, and the ability to expand considerably in hypotonic environments, in contrast to other mammals (15–17). The camel RBCs persist in a shrunken state, retaining their ellipsoidal morphology during dehydration and at elevated temperatures (~40 °C to 45 °C). Upon rapid rehydration, the intravascular compartment transitions to a hypotonic milieu, and RBCs swell, adopting a more spherical form, but do not lyse. The RBCs have a special membrane composition, which protects them from fusing with the membrane proteins of nearby RBCs, which probably explains why camel RBCs barely agglutinate (14, 16, 18). This indicates that camels primarily retain water in their RBCs and other cellular structures, rather than in their adipose tissue, such as the hump, or in their specialised gastric compartments (19, 20). In the dromedary camel, 7.1–10.9×10^6^/μl RBCs circulate in the blood (21). RBCs of camels are ellipsoidal and with tropical waist, anucleate with a long axis diameter of 7.0-10 µm and 4-6.5 µm in short axis (22). A tri-laminar plasmalemma of 6.25 nm encloses the cytoplasm of RBCs (23). Most camel RBC membrane proteins are integral, as opposed to human RBC. The protein-to-lipid ratio of RBCs is higher in camels than in humans (24). Changes in the major integral proteins and the distribution of the glycoproteins in the cell membrane explain the high resistance to osmotic pressure in camel RBCs. Marginal bands are provided with a microtubular system. They are involved in the maturation process and the early maintenance of cell shape, which, in the case of camelid RBC, is ellipsoidal or oval (25). Camel RBCs appear to be resistant to complement-mediated hemolysis (26).

### White blood cells (leukocytes)

The WBCs of camelids are similar to those of other mammals. Neutrophils have a characteristic segmented nucleus and may display subtle pink to purple stippling in their normal cellular structure. The neutrophil-to-lymphocyte ratio (NLR) in adult llamas and camels is approximately 5:1 aligning more closely with humans, dogs, cats, and horses than ruminants (27–29). The NLR has been observed to be lower (0.5–2.9) in a cohort of 29 alpacas; however, the alpacas were aged 10–18 months, and the variation may be attributable more to age rather than to species differences (30).

The WBC count in dromedary camels appears to be in the range of 8.3-19.6 x10^3^/µl blood. Approximately 60-70% of the leukocytes are neutrophils, followed by lymphocytes (31, 32). The neutrophil nuclei of healthy camels show significant lobulation, predominantly 5 lobules per nucleus, characterised by both euchromatin and heterochromatin regions, alongside a notably low nucleus-to-cytoplasm ratio. Additionally, the surface of the neutrophils displays numerous pseudopods. The cytoplasm contains substantial granules of varying sizes and shapes, exhibiting high density centrally, as well as an excess of organelles, including abundant mitochondria, rough endoplasmic reticulum, microtubules, phagolysosomes, vacuoles, and the Golgi apparatus (33) (**Figure 1B**).

Dromedary camel neutrophils appear as highly complex granular cells that express CD45 and CD172a. Within the granulocyte population, dromedary camel eosinophils can be distinguished from neutrophils by their greater green autofluorescence (34, 35).

A group of cell adhesion molecules mediate neutrophil adherence to endothelial cells and the ensuing extravasation processes, therefore organizing the process of neutrophil recruitment to inflammatory sites. The integrin, Lymphocyte Function-Associated Antigen-1 (LFA1; CD11b/CD18) and Macrophage Antigen Complex-1 (MAC1; α_M_β_2_; CD11b/CD18), are expressed in dromedary camel neutrophils at comparable levels to human neutrophils (36).

Dromedary camel neutrophils express a modest but noticeable level of the lipopolysaccharide (LPS) co-receptor CD14, like bovine neutrophils (37), which may indicate a function in Gram-negative bacterial sensing. In a whole blood stimulation study, LPS derived from *Escherichia coli* caused dromedary camel neutrophils to get activate and degranulate. Furthermore, phagocytic activity of dromedary camel neutrophils was decreased by LPS activation; however, their capacity to produce reactive oxygen species (ROS) was unaffected (35).

Young dromedary camels, of about 6 months to 1 year age, have more WBCs than adults, with more lymphocytes and eosinophilic granulocytes, but fewer neutrophilic granulocytes (34, 35, 38–40). Neutrophils are the main innate immune cells that quickly arrive at the infection site. They eliminate the pathogen by phagocytosis, ROS, degranulation, and Neutrophil Extracellular Trap (NET)osis (41). Hussen et al. analyzed neutrophils via flow cytometric detection of the granular enzyme myeloperoxidase (MPO), a marker of NETosis, and showed that *T. evansi-*stimulated neutrophil upregulated MPO expression (39).

Eosinophil counts are generally higher in healthy adult llamas compared to other species (27, 40). Eosinophils typically exhibit hyposegmentation, characterised by band-shaped to bilobed nuclei (**Figure 1B**). There are considerable fine, spherical eosinophilic granules in the cytoplasm, a matrix that holds them together. Basophils can sometimes be seen in the peripheral blood smears of healthy camelids. They are easy to spot since they have plenty of granules that can cover the banded nucleus (27, 40).

The eosinophil granules of the dromedary camel show significant variation, ranging from being round to ellipsoidal or rhomboid shapes, with dimensions of 0.4–2.3 µm in length and 0.3–1.0 µm in width. The individual granule has an electron-dense crystalloid core surrounded by a lighter, homogenous matrix. The crystalloid cores vary in size and shape, frequently appearing segmented and displaying diverse lamellated patterns that are transverse, longitudinal, or concentric to the core’s long axis. It is common to find many crystalloid cores inside a single granule. The pronounced variability of individual granules and the diversity of lamellated patterns distinguish eosinophils of dromedary camels from those of other animals (42).

Healthy camelids generally have low monocyte counts. Monocytes share morphological characteristics with other species, including thick band nuclei and coarse chromatin. As the cell matures during immune response, the nuclei may become rounded containing numerous vacuoles (43).

The fine structure of dromedary camel monocytes reveals small cisternae of rough endoplasmic reticulum and numerous vesicular forms associated with the Golgi complex, positioned around the mitochondria proximal to the primary indentation in the nucleus. Three subpopulations of monocytes in dromedary camels have recently been identified based on the expression patterns of CD172a, CD14, MHC class I and CD163 (36), although there are no monoclonal antibodies cross-reactive with camel CD163 (44). Like the porcine and bovine systems, the signal-regulatory protein alpha (CD172a) has been found to be a pan-monocyte marker for camel monocytes. Classified as camel monocyte cMo-I, the most prevalent subgroup of camel monocytes (87% of total monocytes) has high levels of CD14 and CD163 but low levels of MHC class II. A second fraction of camel monocytes (approximately 6% of all monocytes) is characterised by high levels of CD14, CD163, and MHC class II and is designated as cMo-II. The third monocyte subpopulation, cMo-III, accounts for approximately 5% of all monocytes. It has high levels of MHC class II but low levels of CD14 and CD163 (35, 36) (**Figure 1C**). These distinctive monocyte cell surface signatures probably allude to their division of labor for phagocytic and antigen presenting (following macrophage differentiation) functions.

### Natural Killer cells

In a recent study, gene families that encode the camel NK cell receptors, the natural killer complex (NKC) and the leukocyte receptor complex (LRC), which mediate their function through the interaction with MHC class I molecule, were analyzed. The diversity, content, and organization of the two diverse genomic regions, LRC and the NKC, and the natural cytotoxicity receptor (NCR) genes, NCR1, NCR2, and NCR3, for *C. dromedarius* and *C. bactrianus* species were examined. The camelid NKC extends regionally across a gene structure of ~0.9 Mbp on chromosome 34 and LRC ~0.7 Mbp on chromosome 9. The study revealed a low polymorphism of the killer-cell immunoglobulin-like receptors (KIR) genes in camels, just like the polymorphism of this complex in the pig. In addition, there are considerable differences in genomic organization, polymorphism in LRC of genes encoding NK cell receptors between camels and cattle. The respective protein sequences of the NCR1, NCR2, and NCR3 in the dromedary camel, interpreted as human orthologs, were identified. Only NCR1 and NCR2 are functional genes; NCR3 is a pseudogene. Like cattle, KIR genes, particularly KLRC and KLRH expansions, are not found in dromedary camels. Cattle also express numerous and functional KIRs, while in camels, the KIR locus is represented by a single pseudogene (45). The roles of camelid NK cells in defense against viral infection via interferon production and in fetal-maternal interaction are yet to be ascertained.

### Macrophages

In healthy dromedary camels, the tracheal wash (TW) fluid contains mostly macrophages (51.6 ± 10.2%). The size of camel alveolar macrophages in both TW and bronchoalveolar lavage fluid (BALF) samples varies somewhat, and their cytoplasm includes abundant vacuoles and uneven cell edges (**Figure 1C**). Cellular debris can be found in the cytoplasmic vacuoles of macrophages (46). The initial study employed flow cytometry to examine the immune cell composition of BALF in dromedary camels. The expression of cell surface markers, CD172a, CD14, CD163, and MHC class II molecules in BALF cells indicated that myeloid cells in the BALF of dromedary camels have a phenotype similar to cattle (cows) and pigs. Examination of the cellular composition of the BALF from dromedary camels with bacterial or viral lung infections revealed that the affected camels had a higher total cell count, more neutrophils, and a reduced number of macrophages relative to their healthy counterparts. The presence of elevated MHC class II molecules on alveolar macrophages from diseased camels indicated a propensity towards an inflammatory macrophage phenotype (M1) (47), and readiness for antigen presentation. However, M1 and M2 phenotypes and polarization mechanisms are yet to be established in camelids.

## Lymphoid organs in camelids

The lymphoid organs of camelids differ from ruminants in terms of gross anatomy and histology, particularly the thymus, tonsils, and Peyer’s patches (PPs) (48). The lymphoid organs are classified as central and peripheral. The central lymphoid organs are where the lymphocytes are generated and undergo maturation or differentiation.

### Central lymphoid organs

#### Thymus

The thymus of the dromedary camel is situated in the thoracic cavity, specifically in the anterior superior mediastinum, anterior to the heart and posterior to the sternum. It may extend to the posterior portion of the neck, where it is located between the trachea and the left external jugular vein (49). Each lobe is encased in a delicate connective tissue capsule from which slender connective tissue septa extend, partially partitioning the lobe into lobules. The thymic lobules have an uneven contour and comprise an outer cortex and an inner medulla. The cortex is intensely stained because of the presence of numerous T-lymphocytes (thymocytes). In addition to thymocytes, there are epithelial reticular cells, which are stellate in structure and far less in number than thymocytes (50). Moreover, variations in size and shape of numerous granular cells have been noted in the interlobular septa, occasionally present close to the cortex (51).

#### Bone marrow

The cellularity of normal camelid hematopoietic (red) bone marrow is comparable to or slightly expanded relative to other mammals. A study involving 7 llamas and 20 dromedary camels has indicated a cellularity range of 50 to 85%. Sternal bone marrow aspiration from llamas and rib bone marrow from dromedary camels were examined (52, 53). The morphology and maturation of erythroid cells in the bone marrow were comparable to other species in both llamas and dromedaries. Erythroid cells transitioned from a round to an ellipsoidal shape as their polychromatophilia decreased. No dacryocytes, spindle-shaped RBCs, or uneven hemoglobin distribution were observed in the bone marrow samples (52). The camelid RBCs acquire their ellipsoidal shape during maturation of reticulocytes into erythrocytes, with a circumferential bundle of microtubules, called the marginal band (52, 54, 55). No distinctive characteristics of WBC morphology or maturation have been documented in camelid bone marrow samples. Despite its obvious significance, the camelid bone marrow has not been extensively characterized.

### Peripheral lymphoid organs

#### Mucosal associated lymphatic tissue (MALT)

##### Gut-associated lymphoid tissue

Bactrian camels possess a specialized intestinal mucosal immune system that enables them to combat infections in their harsh environments. The abomasum, one of the three stomach compartments in the Bactrian camel, possesses distinctive structures of MALT (56–58). Histological observations indicated that MALT, including PPs, isolated lymphoid follicles (ILF), along with sparsely distributed lymphocytes, predominantly reside in the *lamina propria* and submucosa extending from the ileocecal valve, where the muscularis mucosa is typically deficient, to the ascending colon. A well-developed inductive site for the mucosal immune system within the digestive tract are the scrotiform PPs at the ileocecal valve and adjacent regions of the terminal ileum. The effector sites of the mucosal immune system are T cells and plasma cells sparsely distributed throughout the large intestine mucosa. The scrotiform PPs in the Bactrian camel remain intact even in the harsh conditions where these animals live (59).

##### Bronchus-associated lymphoid tissue (BALT)

BALT mediates the localized immune response in the airways to airborne pathogens. This tissue structure differs depending on the species and age. In the dromedary camel, the BALT ranges from a few lymphocytes to discrete lymphoid follicles with a germinal centre (GC). In young and adult camels, the BALT of the bronchi consists of prominent secondary lymphoid follicles reflecting antigen-driven T cell-dependent B cell maturation, which shrink with advancing age. The BALT of the bronchioles may be induced in response to an immunological reaction, showing significant morphological differences across various ages. High endothelial venules were linked to BALT in the bronchi but not in the bronchioles. Tall pseudostratified columnar ciliated epithelium with goblet cells in the extrapulmonary bronchi changed to pseudostratified columnar ciliated epithelium with mucous-secreting cells in the intrapulmonary bronchi. Towards the bronchioles, the epithelium transforms from simple columnar ciliated epithelium to simple cuboidal epithelium with Clara cells without goblet cells or mucous-secreting cells (60).

##### Conjunctiva-associated lymphoid tissue (CALT)

In dromedary camels, the CALT is found at the palpebral surface of the conjunctiva in the form of several lymphoid follicles that aggregate near the medial canthus and morphologically appear as a single follicle or as a group of two or more nodules (7, 61). Immunohistochemistry revealed that most CD20^+^ B cells were in the follicular area, while CD3^+^ T cells were mostly localised in the parafollicular area (62). The distribution of B and T lymphocytes in the dromedary CALT follicles are as in the PPs of rabbits and baboons (63). A limited number of CD68^+^ macrophages were detected in various regions, which may engulf foreign antigens and apoptotic cells (64). CD20^+^ B cells, the most prominent cellular component of lymphoid follicles, undergo a T follicular helper cell-dependent positive selection process that eventually leads to the the synthesis of high-affinity antibodies following antigen processing and presentation (64). The follicular dendritic cells (FDCs) in the dromedary camel CALT follicles are similar to those in bovine tonsils. FDCs create an extensive three-dimensional mesh, functionally sustaining T lymphocytes within lymphoid follicles (65). The location of B lymphocytes and FDCs in the GCs of dromedary camel CALT follicles indicates that the interactions between B cells and FDCs are crucial for B cell survival and development in the GC (66). CD3^+^ T follicular helper cells located in the lymphoid follicles may function as Treg in T or B-cell-dependent GC regions (67). These may represent T follicular helper cells in Bactrian and dromedary camels, and other species as well (64). Numerous isolated lymphoid follicles are also observed in the bulbar conjunctiva of the third eyelid reflecting an inductive site of conjunctival B cell maturation.

### Tonsils

Achaaban et al. described the histological and anatomical characteristics of three tonsil groups of the dromedary camel. The palatine, lingual, and velar tonsils make up the oropharynx group, whereas the pharyngeal and tubal tonsils constitute the nasopharynx group, and the paraepiglottic tonsil makes up the laryngopharynx group. All the described tonsils, situated in the pharyngeal cavity, serve as the primary defense barrier against invading microorganisms. A detailed examination of the oral cavity, soft palate, and pharynx has revealed morphological characteristics of various tonsils. The lingual, palatine, velar, and para-epiglottic tonsils are arranged in tightly packed groups. Lymphoid follicles have multiple crypt apertures on the mucosa of the oropharynx. Lymphoid cells invade the crypt epithelium, making it easier for them to interact with antigens. The nasopharyngeal tonsils, which are composed of both pharyngeal and tubal parts, have follicles that are not tightly attached and extend into the epithelium above them. The pronounced growth and distinctive configuration of the tonsils in dromedary camels, along with the frequent occurrence of crypt formation within these tonsils, exemplify another aspect of their adaptability and resistance to their challenging environment (68).

### Peyer’s patches (PPs)

In juvenile dromedary camels, the PPs present as cup-shaped bodies elevated approximately one cm above the luminal surface. Each mass is formed as an aggregate of lymphoid follicles situated in the submucosa, with some extending into the *lamina propria*. The follicles are located along the lateral margins and at the base of the PP. The number of follicles per PP varies between 25 and 27 in the cranial (oral versus aboral, proximal versus distal, luminal versus serosal) region, and between 31 and 38 in the caudal region. The diameter of PP varies between 500 and 900 µm. In adult dromedary camels, the quantity and dimensions of the PP are smaller compared with those of young camels (69). PPs are covered by the follicle-associated epithelium, which like in other domestic animals, lack intestinal villi (69, 70).

### Lymph nodes

The lymph nodes of most mammalian species are similar in structure. Those of the dromedary have an outer cortex and an inner medulla enclosed by a capsule consisting of dense connective tissue. The cortex comprises lymphoid follicles, which are groups of B cells, while in the medulla, T cells, plasma cells and macrophages, predominate. The immune system interacts with its microenvironment, which can change the shape of its functional compartments, like the size of follicles and the thickness of the mantle (71). Additionally, the focal discontinuity or thinning corresponds to the regions where the afferent vessel penetrates the organ. It is also important to note the presence of trabecular extensions that extend toward the cortico-medullary zone, forming the lobules that are the morpho-functional zones. These lobules are divided by lines of cortical and medullary sinuses, with a highly variable number of lobules. The para-cortical sinuses are located within these layers. Lymphoid follicles in the mesenteric lymph nodes are described as sigmoid and rounded, while the medullary zone contains a diffuse lymphoid tissue encircled by small medullary sinuses. In addition, the medullary zone occupies a greater surface area than the cortex. The proportion of active follicles in dromedaries kept in a free-roaming breeding system is noticeably greater than those housed with limited freedom of movement (72). A study by Gavrylin et al. describes the locations, numbers, sizes, shapes, and groupings of superficial and deep lymph nodes throughout the camel’s body, compared with those of other domestic mammals. Topologically, the study focused on the prominent lymph node groups in the somatic lymph nodes (parotid, sub-mandibular, superficial cervical, axillary, popliteal) and visceral (medial retropharyngeal, caudal mediastinal, portal, jejunal, medial iliac) lymph nodes of adult dromedary camels. Parotid lymph nodes had the smallest morphometric indices among the somatic nodes of the camel, while visceral lymph nodes were the largest, and the medial retropharyngeal lymph node was the largest among the other visceral lymph nodes. A total of 131 lymph nodes were observed in dromedary camels. Some visceral lymph nodes of the camel, especially caudal mediastinal and medial iliac, are developed to a greater extent, which is probably caused by the higher degree of functional activity of units (73).

### Spleen

The spleen of the dromedary camel has a crescent shape and is situated at the dorsocaudal region of the omentum (49). The spleen is encased in a robust connective tissue capsule covered by mesothelial cells (74). The capsule can be divided into two distinct layers: the outer layer consists of connective tissue, composed of collagen, elastic fibers, and fibroblasts, along with a limited number of smooth muscle cells, while the inner layer is comprised of smooth muscle tissue, reinforced by connective tissue (74, 75). The trabeculae, whether vascular or avascular, extend from the capsule to the parenchyma (75). The vascular trabeculae encompass nerve fibers and arteries, notably devoid of veins, whereas the avascular trabeculae can be further categorized into primary and secondary trabeculae. The principal trabeculae consist predominantly of smooth muscle cells, which are underpinned by reticular, collagen, and elastic fibers. The secondary trabecula consists of smooth muscle interspersed with reticular fibers. The parenchyma of the spleen in the dromedary camel consists of both white and red pulp. The white pulp consists of the periarterial lymphatic sheath (PALS) along with lymphoid follicles (76). The lymphoid follicles exhibit a spherical morphology, occasionally displaying an indentation on one side corresponding to the location of the PALS (75, 76). The predominant cellular components of the follicles in the white pulp are B lymphocytes, designated as the B-dependent zone in the spleen, whereas the PALS is considered the T-dependent zone (77).

## The immune system of camelids

### Innate immunity

The immune system comprises a complex network of cellular and humoral components designed to defend against invading pathogens. While much research has focused on camel immunoglobulins, comparatively little emphasis has been placed on the cellular and soluble components of the camel innate immune system (78). Compared to other animals within the same geographical region, camels have greater tolerance to viral diseases and environmental stressors (2, 79). The innate immune mechanisms are likely to play a role in this extraordinary host-pathogen standoff; however, this aspect is poorly understood.

A number of proteins of known innate immune functions are found in camel milk. These are defense proteins believed to possess bactericidal, and virolytic capabilities (80, 81). By binding to LPS of Gram-negative bacteria and lipoteichoic acid (LTA) of Gram-positive bacteria peptidoglycan recognition protein (PGRP), a pattern recognition receptor (PRR), it plays a critical role in preventing bacterial adhesion and growth. High levels of this important PGRP are present in camel milk. In a mouse model where a lethal dose of LPS (30 mg/kg) was injected intraperitoneally, followed by PGRP (10 mg/kg) immediately after the LPS injection, camel PGRP demonstrated its anti-inflammatory properties by suppressing pro-inflammatory cytokines, such as IL-6 and TNF-α (79).

### Cytokines and chemokines

Cytokines and chemokines function as key molecules of the immune system, modulating innate and adaptive immune responses. Complete cDNA sequences of major Th1 and Th2 cytokines have been identified from Ilama (82), and the Bactrian and dromedary camel (83, 84). Sequence analysis of these cytokines revealed close phylogenetic relationship with porcine, bovine and ovine orthologs. IL-26 is a new member belonging to the IL-10 family of cytokines and acts as a key signaling molecule and a direct antimicrobial agent. The IL-26 gene is functionally inactivated in several mammals, such as mice, rat, horse, and elephant. However, a functional IL-26 gene has been identified in the dromedary camel (85). Compared to other domestic animals like pigs and cattle, studies on camel cytokines have been limited and camelid-specific immune reagents are scarce. Recently, real-time qPCR assays for simultaneous quantification of camelid (both Old and New World) cytokines have been developed based on the cytokine genes cloned and also from draft genome information (86). Although the camelid cytokines show a high level of sequence conservation with mammalian orthologs, the difference in expression pattern may point towards immune tolerance to certain viruses in camelids. MERS-CoV causes severe disease in humans, whereas it is mostly asymptomatic in camels. MERS-CoV re-exposure results in robust Th1 (IL-2, IL-12 & IFN-γ) and anti-viral type I IFN responses; however, the expression of pro-inflammatory cytokines (IL-1β, IL-6, IL-8 and TNF-α) appears dampened in llama lymph node cells (87). This differential modulation of the cytokine response may aid camelids to host such viruses without any severe clinical manifestations of the disease.

*Brucella* infected dromedary camels have elevated levels of IL-1β and IL-10, whereas TNF-α, IFN-γ and IL-1α expression is decreased. The elevated IL-10 level are presumed to downregulate the Th1 response, whereas reduced IFN-γ/TNF-α ratio points towards *Brucella* ability to evade the immune response (88). Dromedary camels vaccinated with *Brucella abortus* RB51 produce a strong Th1 immune response, characterized by elevated levels of IL-6 and TNF-α, suggesting long-term immunity (89). Dromedary camels infected with the protozoan parasite, *Trypanosoma evansi*, also mount a robust innate immune response with significant increase in pro-inflammatory cytokines such as IL-1α, IL-1β, IL-6, IFN-γ and TNF-α. Some of these cytokines are involved in the production of acute-phase proteins and can be useful as biomarkers in monitoring the progress of *T. evansi* infection in camels (90). Pro-inflammatory cytokines, including IL-1α, IL-1β, IL-6, IFN-γ, and TNF-α, are elevated in dromedary camels with clinical endometritis. However, dromedary camels with endometritis have lower levels of the anti-inflammatory cytokine, IL-10, than healthy camels. It is thought that the onset and progression of endometritis are influenced by the imbalance between pro-inflammatory and anti-inflammatory cytokines (91).

IFNs are a group of helical cytokines that function against viral infections. IFNs are grouped into three types (type I, II and III), based on their cell surface receptors. In placental mammals, multiple classes of type I IFNs are found, which include IFN-α, IFN-β, IFN-δ, IFN-ϵ, IFN-κ, IFN-τ, IFN-ω, IFN-ν, and IFN-ζ. Some type I IFNs like IFN-β, IFN-ϵ and IFN-κ are single copy genes. Other type I IFNs such as IFN-α and IFN-δ have multiple subtypes as they have undergone extensive gene duplication during mammalian evolution (92). 11 IFN-α subtypes have been identified and cloned from the dromedary camels. The sequence analysis of these 11 dromedary IFN-α proteins revealed the conservation of essential motifs and five cysteine residues essential for structural integrity and function. *E.coli* inclusion body-derived recombinant dromedary camel IFN-α protein showed functional activity (93). The *IFN-β* gene has been identified and cloned from dromedary camel, the Bactrian camel, and all four New World camelids (alpaca, vicuna, llama and guanaco). Almost all placental mammals have an odd number of conserved cysteine residues (mostly three) in their mature IFN-*β* protein. Camelids seem to be the only eutherian mammals with an even number of cysteines (four) in their mature IFN-β protein (94). IFN-δ are atypical type I IFN that is identified in a few mammals like sheep, pig, and horse. Human and bovine genomes do not seem to encode functional IFN-δ genes. Seven functional IFN-δ subtypes (IFN-δ1 to δ7) and a pseudogene are identified from the dromedary camel. Prokaryotically expressed recombinant dromedary camel IFN-δ1 exhibits typical interferon like activity *in vitro* (95). IFN-ϵ has also been identified in the dromedary camel and the recombinant protein showed cytotoxicity on human breast cancer cell lines (96). Other type I IFN genes, such as IFN-K, IFN-ω etc have been predicted based on some of the draft camelid genomes but are yet to be isolated and characterized. In mammals, type II IFNs are represented by only IFN-γ; it is one of the first identified camelid interferon (83). A comparative study across mammalian genomes predicted multiple IFN-λ (type III IFNs) genes in the dromedary camel genome; however, these IFN-λ genes are yet to be cloned and functionally validated (97). Considering the extensive gene duplication of some of these IFN subtypes and the presence of many pseudogenes within the mammalian genome, elucidation of the complete type IFN gene repertoire of camelids will require high-quality refined genome assemblies and comprehensive comparative gene annotation.

Lymphocytes from the llamas re-exposed to MERS-CoV show a significant increase in type II IFN (IFN-γ) expression. However, the expression of type I (IFN-α and IFN-β) and type III (IFN-λ3) IFN is transient and dependent on the type of virus (87). IFN-β mRNA expression is suppressed in the early stages of camelpox infection. To evade the host immune response, poxviruses like the camelpox virus have developed mechanisms to suppress the IFN system at multiple levels. CMLV055 in camelpox virus is orthologous to E3L in vaccinia virus, which inhibits IFN-β expression by targeting IRF3 transcription factor (94). Intensive and comprehensive transcriptomic approaches may reveal how different IFNs are modulated by different viruses that infect camels.

Chemokines are low-molecular-weight proteins essential for the spatio-temporal coordination of an immune response by regulating the movement of specific subsets of leukocytes to sites of infection. Although multiple chemokine genes have been predicted from the draft genomes of the dromedary camel and alpaca, camelid chemokines have received very little research attention. Dromedary camel CXCL8 is the first camelid chemokine to be identified. CXCL8 (IL-8) acts as a major neutrophil recruiter and activator. Infection with the camelpox virus *in vitro* induced CXCL8 expression in dromedary kidney cells. Camel kidney cells exposed to heavy metal compounds of cobalt and cadmium *in vitro* had elevated expression of CXCL8, suggesting potential use as a biomarker in assessing renal toxicity (98).

### Complement system

Complement plays an important role in bridging innate and adaptive immunity. The concentration of complement components and subcomponents rises during the early stages of infection before falling as the illness becomes more chronic (99). Ouma et al. have demonstrated complement classical pathway activation in *T. evansi*-infected dromedary camels. Immunosuppression, which is extensively documented in animal trypanosomiasis, may result from decreased complement levels in this species (100). The decrease in complement component levels likely suggests complement consumption during trypanosome infection in camels. There seems to be an inverse relationship between parasite load and hemolytic complement levels. The induction of antibody responses is strongly influenced by experimental decomplementation (101). It has been suggested that hypocomplementemia plays a role in the increased susceptibility to secondary infections in trypanosome-infected animals (102).

The classical pathway hemolytic assay using the dromedary camel serum, under controlled settings were assayed under controlled settings (103). The maximum CH_50_ titer was achieved using rabbit RBCs sensitised with goat haemolysin. CH_50_ titers in the range of 109.8 ± 0.68 to 349.7 ± 59 units per ml of camel serum with the mean of 218.4 were observed. The camel complement was ineffective in lysing sheep or goat RBCs sensitised with rabbit or camel hemolysins. Consequently, the camel exhibits a high degree of erythrocyte- antibody compatibility similar to bovines, buffaloes, and other related ruminants (104). However, careful studies on the complement system of camels are lacking.

### Adaptive immunity

Apart from having interspecies adaptations to extreme climatic conditions, camelids also have species-specific characteristics of the adaptive immune system with unique, heavy chain-only homodimeric antibodies (HCAbs), and they also exploit somatic hypermutation in the T cell receptor (TCR) of their *γδ* T cells (5, 6). The genes of Th1 (IL-2, IL-12 and IFN-γ) and Th2 (IL-4, IL-10 and IL-13) cytokines identified in the Bactrian camel (83, 105) may serve as a rich resource for functional investigations on T cell polarization.

*In vivo* cytokine response studies have relied on mRNA expression assessment. Bactrian camels inoculated with a live attenuated *Brucella abortus* S19 vaccine showed an elevated production of the Th1 cytokine, IFNγ, with little or no expression of the Th2 cytokines, IL-10 and IL-4 (105). A recent immunization trial using ovalbumin showed that the increased cytokine expression pattern of Bactrian camel cells was limited to Th2 cytokines (IL-4, IL-10 and IL-13), therefore addressing the humoral immune response and the generation of antigen-specific antibodies (106). Currently, these studies show a similar polarization of cytokine response following vaccinations as for other mammalian species, especially cattle (107, 108).

Members of the *Camelidae* family have unusual adaptive immune features. Camelids are known to have three IgG isotypes: IgG1, IgG2, and IgG3. While IgG1 has a conventional heterotetrameric antibody structure, both IgG2 and IgG3 consist of two heavy chains with no associated light chains; they include variable domain in their heavy chain (V_H_H), while missing the C_H_γ1 domain that is required for pairing with the constant domain of the light chain. Similar heavy chain antibodies HCABs, consisting of an antigen binding Immunoglobulin New Antigen Receptor (IgNAR) domain and five constant domains, are found in cartilaginous fish such as sharks (109). Given their distinct biochemical and structural characteristics, including high pH stability and low molecular weight allowing for greater tissue penetration, V_H_H domains are being used as the next generation therapeutic antibodies. Commonly referred to as nanobodies, V_H_H domains have several benefits for therapeutic use.

To increase their γδ T cell receptor (TCR) diversity, TCR γ (TRG) and δ (TRD) loci undergo somatic hypermutation (SHM) (110). The genomic studies suggest that the camelid SHM is activation-induced cytidine deaminase (AID) dependent, which is also observed in some sharks (5). It is unclear where in the lymphoid organs this SHM occurs; it is unlikely to be in the GC of lymphoid follicles, where B cells undergo SHM. Alpacas also appear to have a subset of γδT cell (Vγ9Vδ2), which recognise phosphoantigens (earlier known only in primates) (111).

### B cells

In healthy dromedary camels, the major population of peripheral blood lymphocytes are B cells (mean 26.6%), closely followed by CD4^+^ T cells (24.6%) and a small proportion of γδ T cells (7.4%) (34). Healthy camels have similar lymphocyte subpopulations to their closest relatives *Lamini*. B cells are also the predominant lymphocyte population in the blood of healthy alpacas (111, 112). The proportion of B cells among blood lymphoid cells in newborn camels is greater than in adult camels (113).

### Classes of antibodies in camelids

In humans, there are five classes of immunoglobulins: IgM, IgD, IgG, IgA, and IgE. Their heavy chains are denoted by μ, δ, γ, α, and ϵ, respectively. The μ and ϵ chains each comprise one variable domain (V_H_) and four constant domains (C_H_1, C_H_2, C_H_3, and C_H_4), while γ, α, and δ chains contain one variable domain (V_H_) and three constant domains (C_H_1, C_H_2, C_H_3). The V_H_ domain associates with the light chain variable domain (V_L_) to form the antigen-binding site (99). A typical IgG antibody has a roughly Y-shaped structure. It consists of two types of protein chains: heavy chains (H) and light chains (L) joined by disulfide bonds. These are of two types: kappa (κ) and lambda (λ) (99).

**Figure 2:**
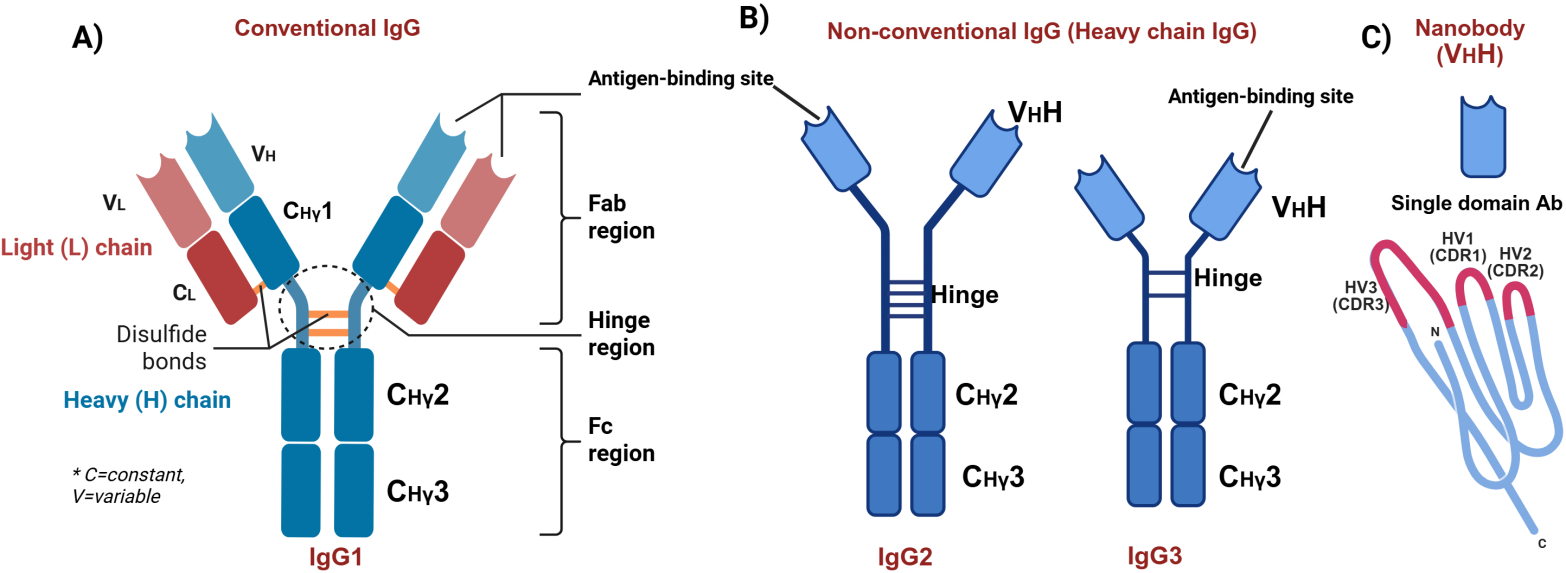
Camelid immunoglobulin G variants: **(A)** Schematic representation of conventional heterotetrameric IgG antibody that has two identical light (L) chains and two identical heavy (H) chains. Each heavy chain has three constant domains (C_H_γ1, C_H_γ2, and C_H_γ3) and one variable domain (V_H_). Each light chain has one constant domain (C_L_) and one variable domain (V_L_). The V_H_ and V_L_ domains come together at the Fab region to make the antigen-binding site. The hinge area allows the Fab and Fc parts move flexibly. Disulphide bonds help keep heavy–heavy and heavy–light chain interactions stable. The Fc region (domains) is responsible for effector actions like complement activation and binding to Fc receptors. **(B)** IgG from camelids (heavy-chain antibodies; IgG2 and IgG3). A diagram showing camelid heavy-chain-only antibodies (HCAbs), which lack the C_H_1 domain or light chains. The antigen-binding site is made up of one variable heavy-chain domain (V_H_H). Inter-chain disulphide linkages stabilise the homodimeric structure of these antibodies, which retain the hinge, C_H_2, and C_H_3 domains. The IgG2 and IgG3 subclasses differ mainly in the length of their hinges and the order of their sequences. The lack of C_H_1 leads to a smaller and more compact antigen-binding structure. **(C)** V_H_H single-domain antibody nanobody. The V_H_H domain is all that is needed to recognise an antigen. Complementarity-determining domains (CDR1, CDR2, and especially the often longer CDR3) make up the antigen-binding interface. Framework regions provide the structure stability. Due to the long CDR3, nanobodies can access cryptic or recessed epitopes that are less accessible to conventional antibodies.

Contrary to human IgG, the humoral immune system of the camelids comprises conventional or canonical heterotetrameric IgG1 (**Figure 2A**) subclass in addition to homodimeric, unconventional or non-canonical IgG2 and IgG3 subclasses (**Figure 2B**) that consist of two identical heavy chains, and are therefore named heavy chain-only antibodies (HCAb) (6), similar to sharks (111). Sequence and structural studies showed many typical characteristics of camelid HCAb that render the H chain functional in antigen binding in the absence of the L chain. The H chain in HCAb is made up of three rather than four globular domains. HCAbs contribute 75% of the total serum IgG (114). HCAb and conventional antibodies are transcribed from the same *IGH* locus where gene duplication events formed the basis for this diversification. ~40 V_H_H genes are used for recombination processes of the antigen binding site (115).

The camelid HCAb possess the following characteristics (1) as there is no C_H_1 domain, the V_H_H domain is directly attached to the hinge region (2) there is a longer hinge in IgG2 and shorter hinge in IgG3 (3) specific conserved amino acid substitutions in the framework region 2 (FR2), primarily at V_H_ positions that interact with the V_L_ in classical antibodies, including Kabat positions 37, 44, 45, and 47; and (4) distinct CDR3 amino acid composition and a wider length distribution for CDR3 compared with the CDR3 in the variable region of the heavy chain in conventional antibodies (6, 116–119). Therefore, in HCAb, the variable region is placed immediately N-terminal to the hinge region (120, 121).

The number of IgG subclasses varies among camelid species. The IgG1 subclass represents classic heterotetrameric antibodies, which show high affinity to protein A and G, and constitute 25% of serum IgG (6, 122). By contrast, the subclasses IgG2 and IgG3 consist of homodimers of shorter heavy chains without the CHγ1 domain. Five IgG subclasses have been reported to be present in dromedaries: two conventional antibodies, IgG1a and IgG1b, and three HCAb, IgG2a, IgG2c and IgG3 (115). Llamas express four subclasses of HCAb, IgG2a, IgG2b, IgG2c and IgG3, and two classic antibody subclasses, IgG1a and IgG1b (117, 119). IgG2 and IgG3 are HCAb and have a molecular mass of 90 kDa. The reduction in mass leads to increased biodistribution and tissue penetration. Long CDR3 loops play an important role because they penetrate deep into grooves at the site of the epitope facilitating the neutralisation of the pathogen (114, 116, 123). The dromedary IgG1 fraction purified from sera by protein A and protein G chromatography (6) contains approximately 3 mg antibody per ml of serum, whereas the HCAb fractions IgG2 and IgG3 contain 1 and 2 mg antibody per ml of serum, respectively (124). The amount of HCAb in llama serum is lower, and that of IgG1 is comparatively higher.

The camelid IGLV framework region shares 65-90% similarity with humans (125, 126). In the context of alpacas, at least three distinct variable (V) gene subgroups are identified within the alpaca IGH locus as described by subsequent studies: IGHV1, IGHV2, and IGHV3. These are homologues of human IGHV families of clan I (VH families 2, 4, 6), clan II (VH families 1, 5, 7), and clan III (VH family 3) respectively, as determined by their sequence homology. Alpaca V_H_H genes are grouped into six groups based on sequence similarity, although all of them are comparable to human IGHV3 genes. Newly discovered camelid classical IGHV genes are closely homologous to human IGHV5 and IGHV7 families (human clan II) in llamas and alpacas, and are from IGHV families 1, 3 and 4 (human clans I and III). Nonetheless, no human gene 2 or gene 6 analog (in humans clan I) was observed (127). A novel promiscuous family of V genes from camelids similar to human V_H_4 family (clan I) was also detected, which can lead to synthesis of both classical tetrameric and homodimeric HCAb. In addition, the V_H_4 homologues are an important part of the classical antibody repertoire; however, they do not have solubilizing V_H_H residues in FR2 that are typical of them (128).

### IgA in dromedary camels

**Figure 3:**
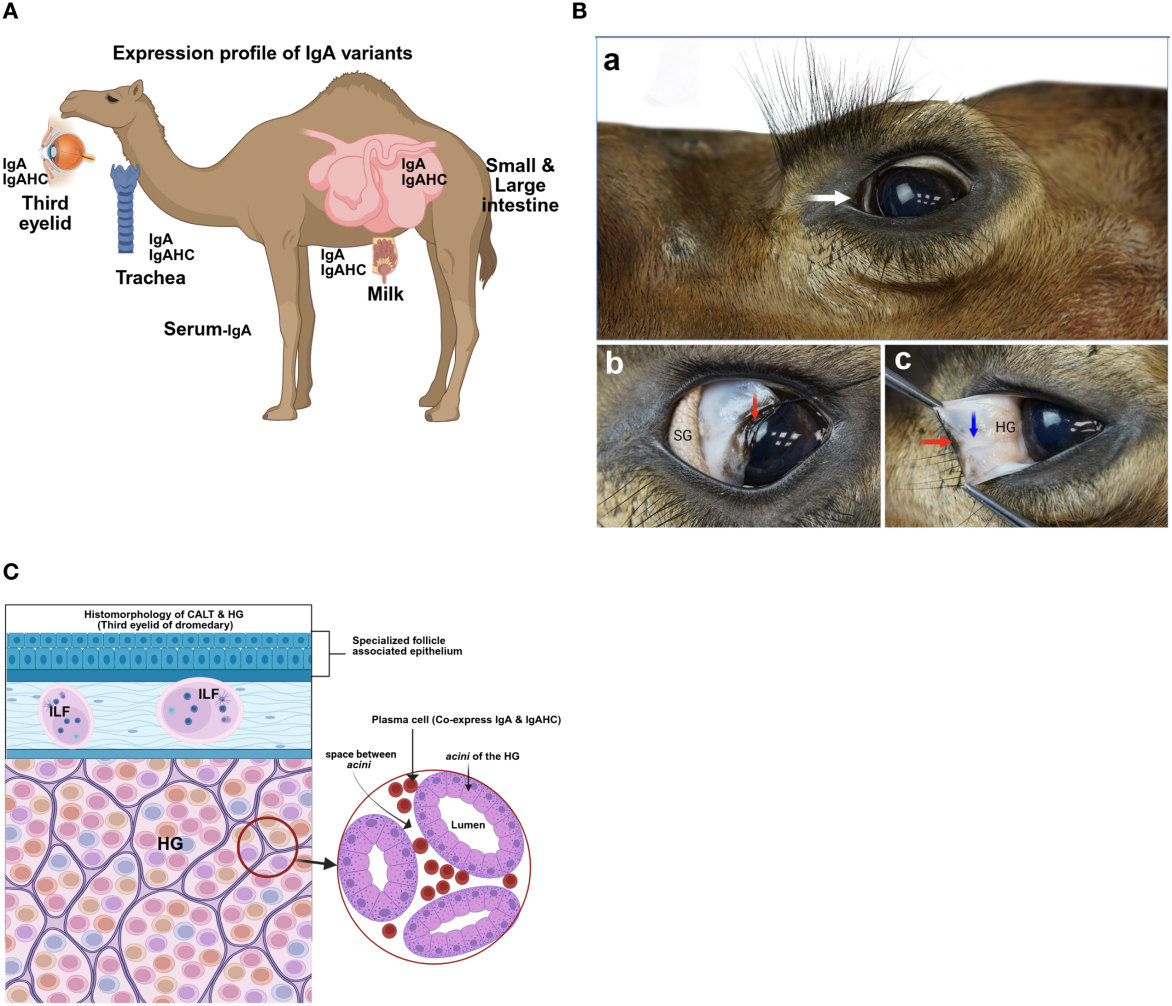
A) Expression profile of IgA variants in camels. A diagram showing where IgA subtypes are found in the dromedary camel’s body. Both conventional IgA and heavy chain-only IgA (IgAHC) are found in mucosal membrane and exocrine glands. Co-expression is shown in the trachea, small and large intestines, and third eyelid (nictitating membrane). The diagram shows that IgA and IgAHC are mostly found in the mucosa, which supports their involvement in barrier immunity at the eyes, respiratory and intestinal. **(B)** Anatomy of the eye, skull and third eyelid of the dromedary. a: close-up of the left eye with retracted third eyelid at the medial angle (arrow). b: detailed view of the third eyelid while blinking. The red arrow indicates the melanin-enriched free margin. The superficial sebaceous gland (SG) is visible at the medial canthus. c: internal surface of the third eyelid. The Y-shaped cartilage shaft (blue arrow) traverses horizontally first the Harderian gland (HG), then the body and ends at the free margin of the third eyelid (red arrow). **(C)** Histomorphology of CALT and Harderian gland (HG) in the dromedary’s third eyelid. The upper panel shows the conjunctiva-associated lymphoid tissue (CALT) beneath a specific kind of follicle-associated epithelium. Isolated lymphoid follicles (ILF) are seen in the submucosa, which shows that the mucosal immune structures are well-organised and may sample antigens and start local immune responses. The Harderian gland (HG), which lies in the third eyelid, is made up of tightly packed secretory acini. A larger inset shows glandular acini around a central lumen, with plasma cells in the gaps between the acini. These plasma cells express both IgA and IgAHC, which suggests that they secrete both conventional and heavy chain-only IgA into the glandular lumen. This supports that the HG is important for the dromedary’s ocular mucosal immune defense.

IgA, the principal immunoglobulin class at mucosal surfaces, consists of conventional and unconventional antibody variants in dromedary IgG. The third eyelid, which like in other quadrupeds, includes a large sebaceous gland, the Harderian (lacrimal) gland and CALT, represents a major source of tear fluid IgA (129, 130) (**Figures 3A, B**). On the inductive site, CALT of the third eyelid consists of specialized follicle-associated epithelium, the subepithelial dome, numerous isolated lymphoid follicles, and IgA-secreting plasma cells (**Figure 3C**).

**Figure 4:**
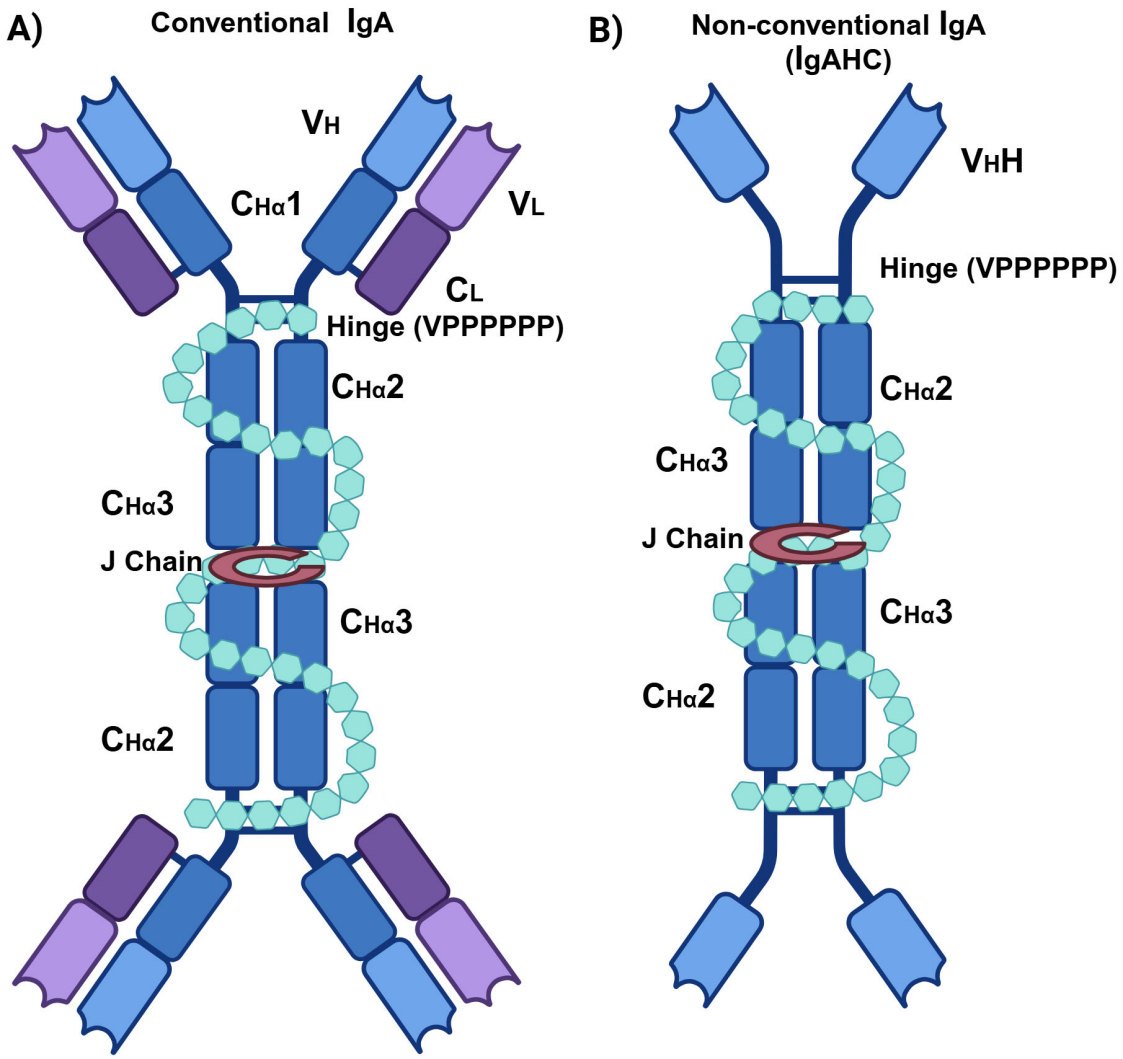
Dromedary immunoglobulin A variants. **(A)** Dromedary IgA. A diagram showing how conventional dimeric IgA is made up of two heavy (H) chains and two light (L) chains. One variable heavy (V_H_) domain and three constant domains, C_H_α1, C_H_α2, and C_H_α3, make up each heavy chain. A light chain has two parts: a variable light (V_L_) domain and a constant light (C_L_) domain. The hinge region (VPPPPPP motif) lets the Fab and Fc regions move flexibly. Dimerization happens when a joining (J) chain connects the C_Hα_3 domains of two IgA monomers. **(B)** Unconventional IgA (IgAHC). Schematic representation of dromedary heavy-chain IgA (IgAHC), which devoid of light chains and the C_H_α1 domain. Only one variable heavy-chain domain (V_H_H) is responsible for antigen binding. IgAHC has C_H_α2 and C_H_α3 domains, and it forms dimers with the J chain. The hinge region (VPPPPPP) gives the structure flexibility. Without light chains, the antigen-binding structure is more compact. Camelid V_H_H domains show characteristic changes in amino acids in their framework regions that make them more soluble and stable even when they are not paired with V_L_. Some important changes are found in FR1 where serine takes the place of leucine and in FR2, phenylalanine turns into valine, glutamic acid turns into glycine, arginine turns into leucine, and glycine turns into tryptophan. These alterations stabilise V_H_ and V_L_ interactions, allowing the V_H_H to fold on its own.

Conventional IgA (1.6 kb cDNA) includes the C_H_α1 domain, like human IgA1 and IgA2 (131); however, the unconventional counterpart (1.3 kb cDNA) does not contain C_H_α1, analogous to the absence of C_H_γ1 in camelid IgG HCAb (**Figures 4A, B**). A series of consecutive prolines form the hinge region which resembles the hinge structure in human IgA2 subclass. In dromedary classic IgA, this hinge region is preceded by the C_H_α1 domain, whereas in unconventional heavy chain-only IgA the V_H_H region is found upstream of the hinge. At the amino terminal end of the C_H_α1 domain, a unique camelid-specific pentapeptide was identified whose function is yet to be undefined. All five amino acids of the inter-α region (IAR) were found exclusively in the *Camelidae* family. Immunohistology with rabbit polyclonal antibodies raised against dromedary C_H_α domains verified that both IgA HC variants were expressed in plasma cells in the Harderian gland, conjunctiva, as well as tracheal and intestinal mucosa. Sequencing of two 5’ PCR products (approximately 1100 bp in length) generated from Rapid Amplification of cDNA Ends (RACE) revealed the presence of classic IgA HC which encompasses the 300 bp C_H_α1 domain. In contrast, all 5’ PCR products of 800 bp length lacked the C_H_α1 domain. This indicates that plasma cells in the effector region of the third eyelid synthesise both forms of IgA HC. The CDR3 region of heavy chain-only IgA is longer compared with other species (132). IgA HC was detected in dromedary milk, but concentration was much lower compared to classical IgA (132). An interesting phenomenon was observed in the *lamina propria* of the small intestine, namely the close proximity of IgA-expressing plasma cells and eosinophils, suggesting an interaction between cells of adaptive and innate immunity (133–135). A vital component of respiratory mucosal immunity, the pharyngeal tonsil, which is situated in the nasopharynx, may effectively protect the body against infections that enter through the upper respiratory tract. In mucosal immunity, IgA and IgG are important effector molecules that perform a variety of immunological tasks (136, 137). IgA antibody-secreting cells (ASCs) are far more common in each part of the pharyngeal tonsils of Bactrian camels than IgG ASCs. This means that IgA ASCs could be the main cells that carry out the immune response in the mucous membranes of Bactrian camels’ pharyngeal tonsils (136). V_H_H domains can get recessed or hidden viral epitopes since they are small and have long CDRs (especially CDR3). V_H_H domains have attracted interest for virus neutralization considering better access to hidden or recessed antigenic sites, improved molecular stability, and tissue penetrance.

To date, no IgM HCAb have been shown to exist. As in most mammals, clonal selection of antigen binding B cells involves those that express IgM (and IgD). This suggests that for camelids, naïve B cells should be found that express HCAb IgG isotypes, and that these can be the basis for clonal expansion.

## Nanobodies

NANOBODY is a registered trademark of Ablynx N.V. (Ghent, Belgium); it is a single domain V_H_H antibody fragment derived from camelid HCAb (6) (**Figure 2C**). The V_H_H fragment has a molecular weight of 15 kDa and dimensions of ~2.5 × 4.0 nm. Nanobody has greater potential to overcome the limitations of conventional monoclonal antibodies, due to their smaller size, better physicochemical stability; they have enhanced solubility and greater penetration through tissues. They are also less immunogenic (138–142).

### Structure of a nanobody

The primary structure of nanobodies is similar to that of the V_H_ domains of conventional antibodies. It consists of four conserved framework regions (FR) and three hypervariable regions (HVR) or CDR, which are responsible for the antigen specificity. These FRs form antiparallel β-strands and β-sheets, which lead to the typical tertiary structure of an immunoglobulin fold. The CDRs stick out from this fold as prominent loops that interact with the surface of the antigen (78, 124, 143). The antigen binding by nanobodies occurs with only three CDRs, in contrast to six CDRs in the heterodimeric organisation of conventional antibodies, and thus, can be produced in large amounts via recombinant DNA technology (115, 117, 144).

### Various applications of nanobodies

The unique characteristics of nanobodies enable their use in multiple fields. Biochemical and structural properties of nanobodies have enabled rapid diagnostic adaptation. By generating nanobody libraries against *Trypanosoma congolense* secretome, active *Trypanosoma* infections were diagnosed with a bivalent nanobody lateral flow assay (145). Nanobodies can be used to develop assays for the detection of biologics and therapeutics. A nanobody-based lateral flow immunochromatographic strip assay for recombinant human IFNα2b has been developed for use in patients undergoing hepatitis B/C treatment (146). Nanobodies can withstand high temperature, and pH which makes them excellent candidates as biosensors. A cardiovascular biomarker, fibrinogen, was detected by nanobody biosensor (147). Biosensors for the use of detecting SARS-Cov-2 and MERS spike proteins were also developed (148). Fluorescently or radioactively labelled nanobodies enable optical, PET, and SPECT imaging. Anti-EGFR nanobody 7D12, labelled with IRDye800CW, showed higher tumor uptake than Cetuximab in murine models, demonstrating nanobodies are superior in targeting potential for epithelial cancers (149).

There are a number of ongoing clinical trials involving nanobodies (**Table 1**). These include (i) MUC1-targeted CAR-T cells with PD1 nanobody MSLN for advanced gynecological solid tumors (NCT06904131) (150); (ii) nanobody CD5-CAR T cell therapy for refractory/relapsed T lymphocyte malignancies (NCT07070323) (151); (iii) nanobody-based Bi-epitope CAR-T cells in relapsed/refractory multiple myeloma (NCT06503107) (152); (iv) tri-specific nanobody (SOA101) in subjects with advanced solid tumours (NCT07055594) (153); and (v) nanobody-based CD5-targeted CAR-T cells for the treatment of relapsed or refractory T-cell acute lymphoblastic leukemia/lymphoma (NCT06874946) (154). An anti-PD-L1 nanobody has been shown to strongly inhibit tumor growth (155). Anti-CTLA-4 nanobodies have shown promise in melanoma models, prolonging survival and reducing tumor growth (156).

**Table 2:** Immunological characteristics of camelids: known and unknown facts.

Category	Known facts	Unknown facts
Innate immunity	Camelid especially dromedary has ellipsoidal RBCs with higher level of integral membrane protein Band 3 (9)	Whether the shape of the RBCs has any role during infection and inflammation
	Higher NLR (27–29); NET formation observed in WBCs (42)	NET-associated protein behavior in antimicrobial spectrum should be characterized
	NK cells receptors (activators and inhibitors) and their functions are partly known (48)	Cytokine profile of NK cells are not known
	PRRs like TLRs response during pathogen entry have been studied (93)	Ligand specificity and signaling pathways are not defined
Adaptive immunity	Camelids possess both conventional (IgG1), and heavy chain-only (IgG2, IgG3) antibodies (81)	Their functional differences during infections; Mucosal IgA system is poorly understood How and where HCAb expressing B cells are selected
	Different T cell populations such as CD4^+^, CD 8^+^, and γδ T cells are identified (34);	Roles of γδ T cells in antigen recognition are not studied; Treg and Breg cells are not characterized
	The classical pathway hemolytic complement of the dromedary camel, under controlled settings were assayed (103)	Extensive studies on the complement system of camels are lacking.

Caplacizumab is an FDA-approved nanobody, developed for the treatment of acquired thrombotic thrombocytopenic purpura (aTTP) (157). In Japan, Ozoralizumab, targeting TNF-α, has been approved for the treatment of rheumatoid arthritis (158). Vobarilizumab is a promising candidate nanobody for the treatment of rheumatoid arthritis, targeting the IL-6 receptor (159). The treatment of *Campylobacter jejuni* infections using a nanobody candidate, LMN-101 (159), produced in a spirulina platform, is currently undergoing Phase II clinical trial (160).

Due to their small size, stability, shelf life, and tissue penetration, nanobodies are an attractive alternative to horse-based snake anti-venom antibodies. Nanobody-based antivenoms have less adverse immune reactions compared to traditional antivenoms. Nanobodies against venom toxins from 18 different African snakes were generated by immunization of alpaca and llama. Eight of these high affinity nanobodies were combined to create an antivenom cocktail. This nanobody cocktail prevented experimental *in-vivo* lethality and reduced dermo-necrosis across 17 African venomous snakes, more effectively than conventional antivenom (161).

Although nanobodies have many advantages over conventional antibodies, their small size and unique structure present other set of challenges. The small size of nanobodies (15 kDa) is well below the glomerular filtration threshold (50–60 kDa). Hence, they are rapidly cleared from the blood by the kidneys, resulting in a short serum half-life. To address this, nanobodies are conjugated with anti-albumin nanobody to create multivalent nanobodies that bind to albumin protein in serum to form a larger protein complex above the renal filtration threshold. This binding with albumin targets the nanobody-albumin protein complex to the FcRn recycling mechanism which further extends the serum half-life of the nanobody (162, 163). Some nanobodies like the ^68^Ga-labeled anti-HER2 used in molecular imaging have been found to accumulate in the kidneys. Altering residues or tags at the C-terminus of the nanobody reduces the kidney uptake significantly (164).

Given the immunological and pharmacological advantages and the versatility of nanobodies in diagnosing and treating diseases, combined with cost-effective production processes, it is likely that their clinical application will expand fast and possibly replace some treatments based on conventional monoclonal antibodies.

## Conclusions and perspectives

Camelids possess a specialized immune system characterised by unique evolutionary adaptions that enable survival in extreme environments. These species also exhibit remarkable resistance to various infectious diseases, including MERS-CoV, foot and mouth disease and tetanus. The main adaptations include the existence of heavy chain-only antibodies, SHM of γδ T cells, the presence of potent anti-microbial peptides such as PGRP in milk, along with significant anatomical differences in lymphoid organs compared to ruminants. However, many areas of camelid immunology remain underexplored. There is still work needed to understand how HCAb producing B cells are selected, and whether they represent a lineage of B cells that are distinct from conventional antibody producing B cells. Future research must address understanding innate immune mechanisms including collectins, complement subcomponents and regulators, acute phase reactants and other humoral factors. These studies would be facilitated by the development of more camelid specific monoclonal antibodies. The degree of information across various camelid family members with respect to immune system has been summarized in **Tables 2** and **3**. Unlike bovine or porcine genomes, the existing camelid genomes are not highly refined or comprehensively annotated. This hinders accurate identification and comparative analysis of complex immune gene families. A considerable lack of reagents specific to camelids has affected serious progress in drawing parallels between mammalian and camelid innate and adaptive immune systems.

**Table 3:** Immune features across different camelids species.

Features	Dromedary *C. dromedarius*	Bacterian camel *C. bactrianus*	Llama *L. glama*	Alpaca *V. pacos*
Heavy chain-only IgG (HCAb) IgG2 & IgG3 lacking C_Hγ_1 domain and light chains	Present	Present	Present	Present
Heavy chain only IgA	Present (132)	Absent	Unknown	Absent
γδ T cells somatic hypermutation	Present (5)	Unknown	unknown	Not well defined
NK cell receptor diversity	Not well defined	Unknown	Unknown	Unknown
TLR mediated response	Sequence analysis data is available	Sequence analysis data is available (165)	Unknown	Unknown
Innate/adaptive bridging — type I/II/III IFN responses	Well defined (87, 97, 98)	Well defined (166)	Well defined (87)	Defined (167)
Complement system	Hemolytic activity defined (103)	Unknown	Unknown	Unknown
Monocyte (classical/nonclassical)	Well characterised (46)	Unknown	Unknown	Unknown

**Table 1:** Diagnostic/therapeutic application of nanobodies.

Usage	Application/ disease area	Target molecule	Nanobody/ therapy name	Reference/clinical trial number
Diagnostic	*Trypanosoma congolense* detection*/*Parasitic	*T*. *congolense* pyruvate kinase (*Tco*PYK)	Nb pair (Nb44/Nb42)	(145)
Diagnostic	Estimation of recombinant human interferon α2b/Viral disease	rhIFNα2b	Nb I22	(146)
Diagnostic	Determination of fibrinogen in plasma/Cardiovascular	Human fibrinogen (Fib)	anti-Fib Nb	(147)
Diagnostic	single-molecule detection of SARS-CoV-2 and MERS-CoV antigens/Viral disease	RBD of SARS or MERS coronavirus spike protein	VHH04/VHH72 Nbs	(148)
Diagnostic	preclinical diagnostic imaging of EGFR/Cancer	Human EGFR	7D12-IR	(149)
Immunotherapy/ Cancer	PD1 CAR-T immunotherapy for gynaecological solid tumours	Human PD-1	MUC1-targeted CAR-T cells with PD1 nanobody MSLN dual-target (BZE2203)	NCT06904131 (150)
Immunotherapy/ Cancer	CD5-Nanobody - CAR-T therapy for lymphocyte malignancies	Human CD5	CD5-Nanobody - CAR-T	NCT07070323 (151)
Immunotherapy/ Cancer	BCMA nanobody CAR-T therapy for Relapsed/Refractory Multiple Myeloma	Human BCMA	BCMA Nanobody	NCT06503107 (152)
Immunotherapy/ Cancer	tri-specific antibody against advanced solid tumors	Human PD-L1/HLA-G/CD3	Nb-TriTE (SOA101)	NCT07055594 (153)
Immunotherapy/ Cancer	CD5-Nanobody - CAR-T therapy for relapsed or refractory T-cell acute lymphoblastic leukemia/lymphoma	Human CD5	CD5-Nanobody - CAR-T	NCT06874946 (154)
Immunotherapy/ Cancer	PD-L1 nanobody induce T-cell responses and inhibit tumor growth	Human PD-L1	PD-L1 nanobody (KN035)	(151, 155)
Immunotherapy/ Cancer	CTLA-4 nanobodies for melanoma	Human CTLA-4	Nb16	(156)
Immunotherapy/ Autoimmune diseases	treatment of acquired thrombotic thrombocytopenic purpura	Human von Willebrand factor A1-domain	Caplacizumab (Cablivi)/ALX-0081)	(157)
Immunotherapy/ Autoimmune diseases	trivalent anti-TNFα nanobody for rheumatoid arthritis	Human TNFα	Ozoralizumab (Nanozora)/ATN-103	(158)
Immunotherapy/ Autoimmune diseases	IL-6R nanobody for rheumatoid arthritis	Human IL-6R	Vobarilizumab/ALX-0061	(159)
Immunotherapy/ Infectious diseases	Treatment of enteric campylobacter infection	*Campylobacter jejuni* FlaA flagellin	LMN-101 produced in Spirulina	(160)
Immunotherapy/ Snakebite Antivenom	Nanobody cocktail as Antivenom for 17 African snakes	Elapid snake venom toxins	eight oligoclonal mixture	(161)

The study of camelid immunity has been critical for the development of nanobody-based diagnostics and therapeutics for a range of conditions. Additionally, as climate change increases global environmental stress, understanding these adaptions will provide a vital paradigm for inter-species resilience and the protection of human health from zoonotic threats.
